# Comparative Experimental Infection Study in Dogs with *Ehrlichia canis*, *E*. *chaffeensis*, *Anaplasma platys* and *A*. *phagocytophilum*

**DOI:** 10.1371/journal.pone.0148239

**Published:** 2016-02-03

**Authors:** Arathy D. S. Nair, Chuanmin Cheng, Chanran K. Ganta, Michael W Sanderson, Arthur R. Alleman, Ulrike G. Munderloh, Roman R. Ganta

**Affiliations:** 1 Center of Excellence for Vector-Borne Diseases, Department of Diagnostic Medicine/Pathobiology, College of Veterinary Medicine, Kansas State University, Manhattan, Kansas, United States of America; 2 Kansas State Veterinary Diagnostic Laboratory, Department of Diagnostic Medicine/Pathobiology, College of Veterinary Medicine, Kansas State University, Manhattan, Kansas, United States of America; 3 Department of Physiological Sciences, College of Veterinary Medicine, University of Florida, Gainesville, Florida, United States of America; 4 Department of Entomology, University of Minnesota, St. Paul, Minnesota, United States of America; Washington State University, UNITED STATES

## Abstract

Dogs acquire infections with the *Anaplasmataceae* family pathogens, *E*. *canis*, *E*. *chaffeensis*, *E*. *ewingii*, *A*. *platys* and *A*. *phagocytophilum* mostly during summer months when ticks are actively feeding on animals. These pathogens are also identified as causing diseases in people. Despite the long history of tick-borne diseases in dogs, much remains to be defined pertaining to the clinical and pathological outcomes of infections with these pathogens. In the current study, we performed experimental infections in dogs with *E*. *canis*, *E*. *chaffeensis*, *A*. *platys* and *A*. *phagocytophilum*. Animals were monitored for 42 days to evaluate infection-specific clinical, hematological and pathological differences. All four pathogens caused systemic persistent infections detectible throughout the 6 weeks of infection assessment. Fever was frequently detected in animals infected with *E*. *canis*, *E*. *chaffeensis*, and *A*. *platys*, but not in dogs infected with *A*. *phagocytophilum*. Hematological differences were evident in all four infected groups, although significant overlap existed between the groups. A marked reduction in packed cell volume that correlated with reduced erythrocytes and hemoglobin was observed only in *E*. *canis* infected animals. A decline in platelet numbers was common with *E*. *canis*, *A*. *platys* and *A*. *phagocytophilum* infections. Histopathological lesions in lung, liver and spleen were observed in all four groups of infected dogs; infection with *E*. *canis* had the highest pathological scores, followed by *E*. *chaffeensis*, then *A*. *platys* and *A*. *phagocytophilum*. All four pathogens induced IgG responses starting on day 7 post infection, which was predominantly comprised of IgG2 subclass antibodies. This is the first detailed investigation comparing the infection progression and host responses in dogs after inoculation with four pathogens belonging to the *Anaplasmataceae* family. The study revealed a significant overlap in clinical, hematological and pathological changes resulting from the infections.

## Introduction

During summer months, dogs are likely to acquire infections with many different tick borne pathogens due to increased tick activity. The infections in dogs may include five *Anaplasmataceae* family pathogens; *Ehrlichia canis*, *E*. *chaffeensis*, *E*. *ewingii*, *Anaplasma phagocytophilum* and *A*. *platys* [1,2]. The primary host cell tropism for these pathogens is mononuclear leukocytes for *E*. *canis* and *E*. *chaffeensis*, granulocytes for *E*. *ewingii* and *A*. *phagocytophilum* and platelets for *A*. *platys* [3,4]. Infections with these pathogens can cause clinical and/or subclinical outcomes. Clinical signs induced by these pathogens are often overlapping and may include mild to acute fever, anorexia, lameness, lethargy, lymphadenopathy, hemorrhagic disorders and neurological signs [5]. Infections often persist for long periods of time without clinical manifestations [5]. Recent serological assessment studies in the USA, focused on canine tick-borne diseases, revealed that the pathogens are more prevalent over a wide geographic area [6,7]. Infections with *E*. *canis*, *E*. *chaffeensis*, *E*. *ewingii*, *A*. *platys* and *A*. *phagocytophilum* have also been documented in people [8–11]. *E*. *chaffeensis* and *A*. *phagocytophilum* also infect several other vertebrate hosts [12]. Experimental infection studies have been performed to assess the disease progression and persistence in dogs with *E*. *canis* [13,14], *E chaffeensis* [15], *A*. *phagocytophilum* [16] and *A platys* [17]. To date, however, comparative infection studies focused on assessing the pathophysiological outcomes of multiple tick-borne rickettsial pathogens have not been conducted.

In this study, experimental infections were performed with four different pathogens of the genera *Ehrlichia* and *Anaplasma* in purebred beagle dogs and the infection progressions were followed for 42 days to assess similarities and pathogen specific differences in clinical, hematological, and pathological changes.

## Materials and Methods

### Infection inocula

*E canis* and *E chaffeensis* organisms were cultured *in vitro*, and inocula were prepared and used for infections as we recently described for *E*. *chaffeensis* infections experiments [18]. Briefly, bacteria cultured in the canine macrophage cell line, DH82 with about 80% infectivity were recovered by centrifugation and washing the cell pellets with phosphate buffered saline (PBS). The final recovered cell pellets were resuspended to a concentration of 2x10^8^ *Ehrlichia* organisms per ml of PBS and used for intravenous (IV) infections (1 ml each per animal). The IV inoculum for *A*. *platys* was 1 ml of blood from an infected dog. The sample was collected in EDTA tubes from a clinically ill German shepherd dog at the University of Florida Veterinary Hospital in 2005 and was frozen with 15% DMSO at -80°C until use. *A*. *platys* infection in this animal was confirmed by blood smear examination and by pathogen specific PCR. The *A*. *phagocytophilum* dog isolate named Martin was cultivated in ISE6 tick cells and the inoculum was prepared similar to the *E*. *chaffeensis* inoculum described in [18].

### Experimental infections in dogs

Experiments with dogs complied with the Public Health Service (PHS) Policy on the Humane Care and Use of Laboratory Animals, the US Department of Agriculture’s (USDA) Animal Welfare Act & Regulations (9CFR Chapter 1, 2.31), and were performed with approval of the Kansas State University (KSU) Institutional Animal Care and Use Committees (IACUC) as per the guidelines of the protocol. Sixteen purebred beagle dogs (4–6 month old of both sexes) were purchased from a Class A USDA vendor (Covance Research Products, Denver, PA) and housed in the indoor climate controlled facility at Kansas State University. Animals were provided a diet of commercially available dry dog food and water ad libitum and all animals were also provided adequate space allowing them to freely move about for regular exercise activity. Both non-infected controls and infected dogs were monitored daily for health and behavioral changes and twice weekly for body temperature and hematological changes initially for three weeks. These parameters were monitored once weekly thereafter until the end of study. The body weights of all the dogs including controls were monitored weekly until the end of the study. No dogs developed a serious complication where they needed additional veterinary care and treatment. However, as a precaution, humane end point protocol was in place. A university veterinarian oversaw veterinary care for all of the animals. At the end of each experiment, all animals were euthanized in accordance with the recommendations of the Panel on Euthanasia of the American Veterinary Medical Association (AVMA). Specifically, commercial euthanasia solution, Fatal-Plus^®^, of volume 0.22 ml/kg containing 86 mg/kg of pentobarbital was administered intravenously.

Before initiating the infection study, all animals were confirmed negative for all pathogens by PCR assessment and/or by cell culture assessment. Four dogs (2 male and 2 female dogs) each were infected with *E*. *canis*, or *A*. *platys* and three dogs were infected with *E*. *chaffeensis* or *A*. *phagocytophilum* (2 males and 1 female). Two dogs that were not inoculated with anything were maintained as uninfected controls. All dogs received diphenhydramine syrup (4 mg/kg body weight) about 30 min before being injected to prevent any possible anaphylactic shock.

All animals were monitored for a period of 42 days following experimental infections for clinical signs and hematological changes, as well as to assess persistence of the infection and antibody responses. Body temperatures were monitored twice a week initially for three weeks and once a week thereafter. Similarly, blood analysis was performed twice a week for the first three weeks and then once a week thereafter. Hematology of control dogs was conducted at weekly intervals. Blood smear examination to detect intracellular inclusions of pathogens was carried out for the samples collected during the first 19 days post infection for animals infected with *E*. *canis*, *E*. *chaffeensis* and *A*. *platys*. At the end of the study, all animals were euthanized and tissue samples were collected for further analysis.

### Hematological analysis

About 2 ml whole blood was collected aseptically in EDTA tubes and used for assessing the hematological parameters, such as the complete blood count, packed cell volume (PCV), hemoglobin concentration (Hb) and for the presence of pathogen specific IgG responses. Blood samples were analyzed within 3 h of collection using a Siemens ADVIA 2120 Hematology system at the Kansas State Veterinary Diagnostic Laboratory (KSVDL) for complete blood counts. This system provides complete data profile of blood samples for the above listed parameters. The hematological values of each infected group were compared with the values of uninfected controls.

Thick blood smear slides were prepared and stained with Wright’s-Giemsa stain also at the KSVDL and shipped at room temperature to the College of Veterinary Medicine, University of Florida for direct smear analysis. The smears were examined manually for *Ehrlichia* and *Anaplasma* inclusions (morulae) within the cytoplasm of WBC or platelets, to assess differential leukocyte counts and for the leukocyte morphology. The percentage of granular lymphocytes observed in each blood smear was then converted to absolute numbers/μl of blood using the corresponding TLC values obtained from the CBC analysis.

### Evaluation of blood samples for infection by culture recovery and molecular methods

The infection progression in dogs was monitored by culture isolation and by nested PCR for *E*. *chaffeensis* and *E*. *canis*. *A*. *platys* and *A*. *phagocytophilum* infections were monitored only by PCR. Culture isolation, genomic DNA recovery from blood samples and nested PCRs were carried out as per the protocol described in Nair et al. [18] with minor modifications. Briefly, buffy coats were recovered from about 2 ml of whole blood after removing the plasma and lysing RBCs and used to perform culture recovery experiments. For nested PCR assays, DNA recovered from equivalent of 2 ml of whole blood was resuspended into 100 μl of TE buffer and 2 μl of the final purified DNA was then used for the first round PCR. PCRs were carried out using primers targeting the 16S rRNA genes of *E*. *canis*, *E*. *chaffeensis*, *A*. *phagocytophilum* and *A*. *platys* by following the methods described in [19–22]. Briefly, the first round PCR was carried out in a 25 μl reaction volume using Platinum Taq DNA polymerase (Life Technologies, Carlsband, CA). The PCRs were performed in a GenAmp 9700 instrument (Applied Biosystems, Foster City, CA). The nested PCRs were performed using 2 μl of 1:100 diluted products from the first PCR and using the nested PCR primer sets. Annealing temperatures for *Ehrlichia* and *Anaplasma* DNAs were 50°C and 55°C, respectively. The extension times were 30 sec and 60 sec respectively for *Ehrlichia* and *Anaplasma* DNAs. Products from the second PCR were resolved on a 1.5% agarose gel to identify specific PCR amplicons [23]. PCR product specificity was further confirmed by sequencing analysis of several randomly selected samples. Stringent protocols and controls were included in all procedures to prevent PCR product contaminations.

### Enzyme linked immunosorbant assay (ELISA)

Quantitative ELISAs were performed to determine the concentrations of total IgG and IgG subclasses, IgG1 and IgG2, in plasma samples collected at weekly intervals till the end point of the study. The ELISAs were assessed using the respective pathogen specific total antigens prepared from the *in vitro* cultured organisms of *E*. *canis*, *E*. *chaffeensis* and *A*. *phagocytophilum* [24]. To measure IgG in the dogs infected with *A*. *platys*, the ELISA assays included heterologous antigens prepared from *A*. *phagocytophilum*. Briefly, the 96-well Immulon 2HB ELISA plates (Thermo Fisher Scientific, Waltham, MA) were coated with the purified respective antigen at a concentration of 20 ng/well using 50 mM sodium carbonate buffer, pH 9.6. One-hundred microliters of each plasma sample starting from day zero (prior to infection) and different days post-infection were diluted 1:50 and were added to antigen-coated wells and incubated for 2 h at room temperature. Similarly, plasma samples from uninfected controls were assessed. The wells were washed thrice with PBS containing 0.05% Tween 20 (PBST) and incubated with HRPO-conjugated goat anti-dog total IgG or IgG2 antibody or sheep anti dog subclass IgG 1 (Bethyl Laboratories, Montgomery, TX) at a dilution of 1:40,000. Unbound secondary antibodies were removed by washing with PBST, and the specific interactions were assessed by color development using TMB (3,3′,5,5′-tetramethyl benzidine) (Calbiochem, San Diego, CA) as the substrate as per the kit protocol (Bethyl Laboratories, Montgomery, TX). For determining the concentrations of total IgG or IgG subclasses, serial dilutions of purified dog reference serum with known concentration of total IgG, IgG1 and IgG2 were added to plates coated with anti-dog immunoglobulins. Concentration of the total IgG and IgG subclasses were determined by linear regression analysis. The ELISAs were performed in triplicate wells and the average values were used for the analysis.

### Necropsy, Histopathology and tissue sample analysis

Tissue samples collected at the end point of the study were evaluated for the presence of gross lesions. Brain, bone marrow, lung, liver, spleen, kidney, heart, skeletal muscle, adrenal glands, lymph nodes and skin were fixed in 10% formalin for 24 h and processed in a Tissue-Tek^®^ VIP^®^ 6 Vacuum Infiltration Processor (Sakura Finetek, CA). The processed tissues were embedded in paraffin and 5 μm thickness hematoxylin and eosin (H&E) sections were prepared based on standard protocols routinely followed at KSVDL. All histopathological observations were evaluated by a board certified anatomic pathologist (CKG). Microscopic lesions were seen mostly in lung, liver and spleen; lesions were graded on a numerical scale of 0 to 3 for each tissue based on the severity and distribution of the lesions in two sections of each tissue measuring approximately 2 cm^2^. The numerical grading scale used is as follows. Lung: 0 = no noticeable inflammation; 1 = rare microgranulomas and small numbers of perivascular inflammation; 2 = occasional microgranulomas and moderate numbers of perivascular inflammation; 3 = frequent microgranulomas and large numbers of perivascular inflammation. Liver: 0 = no noticeable inflammation; 1 = mild periportal inflammation with sinusoidal microgranulomas; 2 = moderate periportal and centrivenal inflammation with sinusoidal microgranulomas and 3 = marked periportal and centrivenal inflammation with sinusoidal microgranulomas. Spleen: 0 = normal periarterial lymphatic sheaths (PALS); 1 = mild PALS hyperplasia; 2 = moderate PALS hyperplasia; and 3 = marked PALS hyperplasia. The inflammatory cells observed often included macrophages and lymphocytes in lung and liver. A combined score was given for each animal from the sum of individual scores for each tissue on a scale ranging from 0–9, where higher score suggests more severe lesions.

At necropsy, spleen, liver, lung, and cervical lymph nodes were also collected and processed for PCR assays. Tissue samples for PCR analysis were transferred to -20°C immediately until use. Total DNA from approximately 20 mg each of tissue sample was isolated using a DNA isolation kit as per the manufacturer’s instructions (Wizard genomic DNA isolation kit, Promega, Madison, WI). Genomic DNA from each sample at the final step of the purification was resuspended in 200 μl of DNA rehydration buffer. Two μl each of this solution was then used for nested PCR analysis as described above.

### Statistical analysis

Hematology data for days where control dogs were sampled were evaluated by a nested repeated measures ANOVA of each hematologic parameter accounting for treatment group, day and treatment group by day interaction. For models of hematology outcome with a significant effect of treatment group (p<0.05), individual ANOVA models were evaluated for each day and if the treatment group effect was significant, day specific contrasts of the treatment groups to the control group were performed using a Bonferroni adjustment to account for multiple comparisons. Treatment group to control comparisons were considered different if the Bonferroni p value was less than 0.05. Commercial software (Stata Corp LP, College Station, TX) was used to perform these analyses. A two-tailed unpaired Student’s *t* test was performed for ELISA results using statistical software (GraphPad software, http://www.graphpad.com/, La Jolla, CA).

## Results

### Clinical findings

Prior to experimental inoculations, all dogs were clinically healthy with normal body conditions and body temperature. The average body temperature of control animals throughout the study and pre inoculation values of all four infected groups was 38.9°C± 0.2. This value was accepted as the normal body temperature for this group of dogs. All dogs infected with the four different pathogens; *A*. *phagocytophilum*, *A*. *platys*, *E*. *chaffeensis* and *E*. *canis*, maintained normal appetite and physical activity, and weight gains were also similar throughout the study period compared to uninfected controls. With the exception of high fever at certain days post infection, none of the infected dogs developed clinically noticeable manifestations of an acute illness. In the *E*. *canis* infected group, persistent, but intermittent, fever was observed in all four dogs, which ranged from 39.3–39.8°C (Table 1). Similarly, high fever in *E*. *chaffeensis* infected group was observed for several post infection days starting from day 9 (Table 1). In the *A*. *platys* group, one dog had persistent fever and the fever was very high at 40.9°C on day 17 and 35 post infection. The body temperature of the remaining three dogs in this group and for the *A*. *phagocytophilum* infected group was normal throughout the study period.

**Table 1:** Body temperature changes in dogs infected with *E*. *canis*, *E*. *chaffeensis*, *A*. *platys* or *A*. *phagocytophilum*.

Inoculum	Dog#	Body temperature (°C)* (Days post infection)
*E*. *canis*	1	39.3 (5), 39.6 (12)
	2	39.6 (12)
	3	39.8 (21)
	4	39.5 (29, 35)
*E*. *chaffeensis*	1	39.7 (12), 39.8 (14, 29, 35), 39.7 (19,21)
	2	39.6 (9,14), 39.7 (21, 42), 40.0 (29, 35)
	3	39.3 (16)
*A*. *platys*	2	39.7 (2), 39.4 (5,7), 39.7 (9), 39.8 (12), 40.1 (14), 40.9 (17), 39.6 (19), 40.9 (35), 39.8 (42)
	
*A*. *phagocytophilum*	All dogs	No change in body temperature noted

*Temperatures above the estimated normal body temperature of 38.9°C± 0.2 is considered fever. The table included temperatures recorded only for days when high fever is observed; all others days, the dogs had normal body temperature.

### Hematology and blood smear evaluation

**Fig 1:**
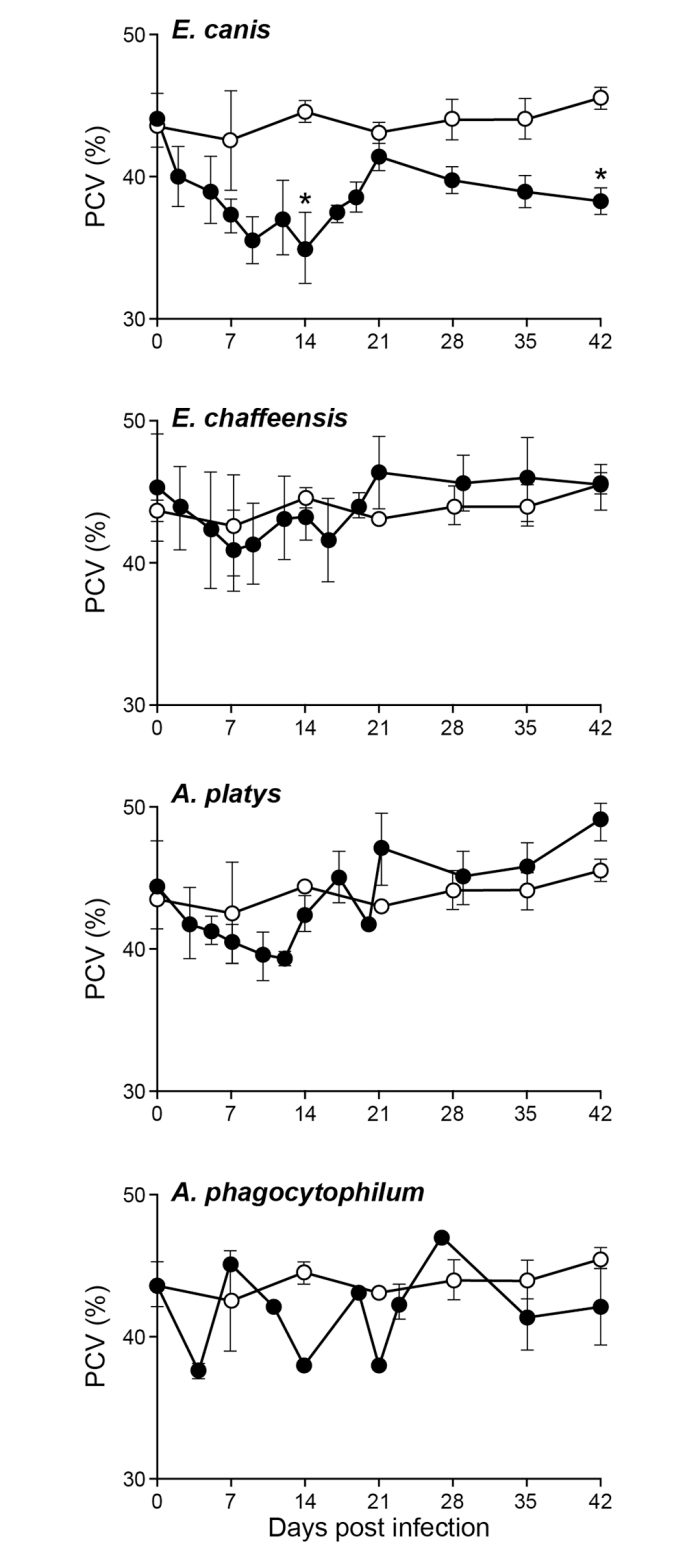
Impact of infections on PCV. PCV values in dogs infected with *E*. *chaffeensis*, *E*. *canis*, *A*. *platys* and *A*. *phagocytophilum* were compared with the values observed for uninfected controls. The values are shown as mean ± SD per group. Significant differences (*P* ≤ 0.05) observed between controls (open circle) and infected groups (closed circle) are identified with asterisks.

**Fig 2:**
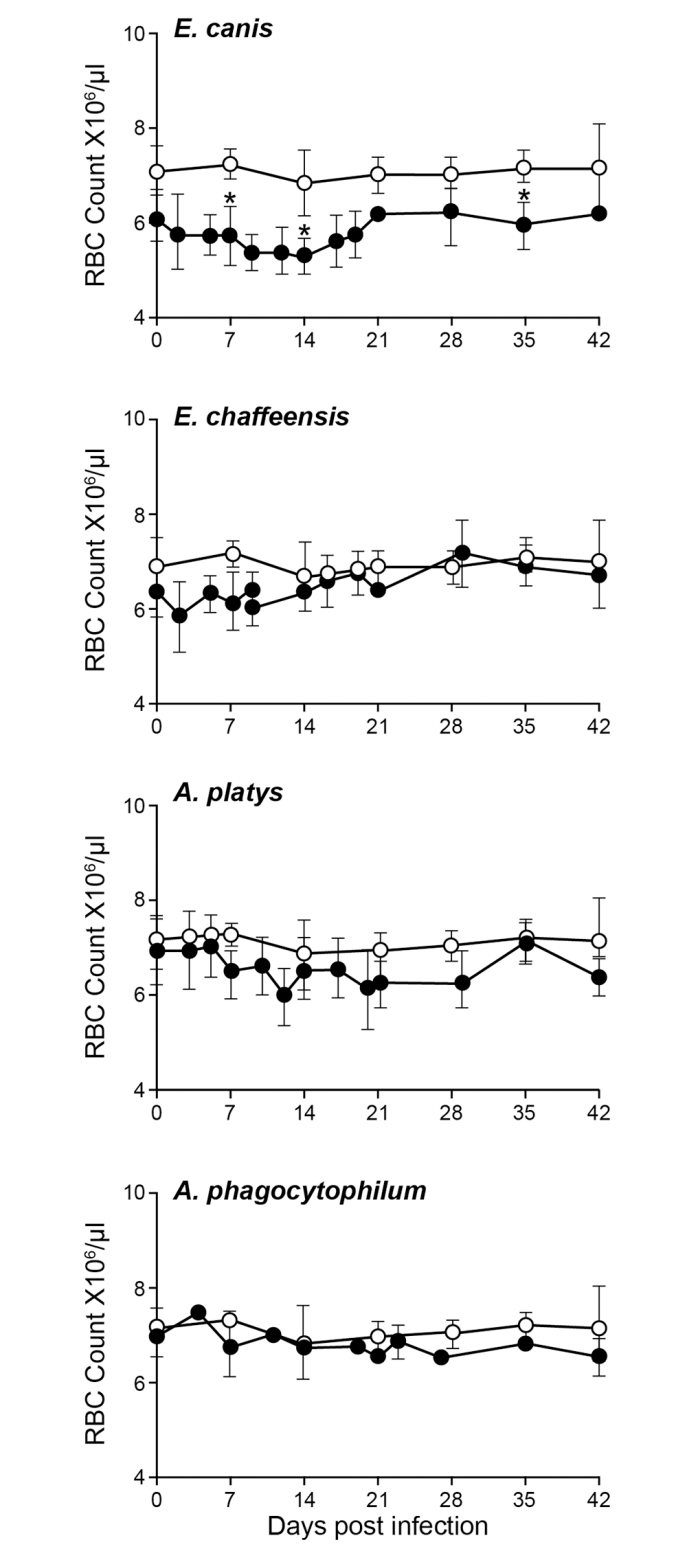
Impact of infections on RBC counts in dogs. RBC numbers in uninfected controls are compared to those observed in infected groups. Error bars for this panel represent mean values ±SD for animals within each group. Significant differences (*P* ≤ 0.05) observed in an infected group (closed circle) compared to non-infected control (open circle) values are identified with asterisks.

**Fig 3:**
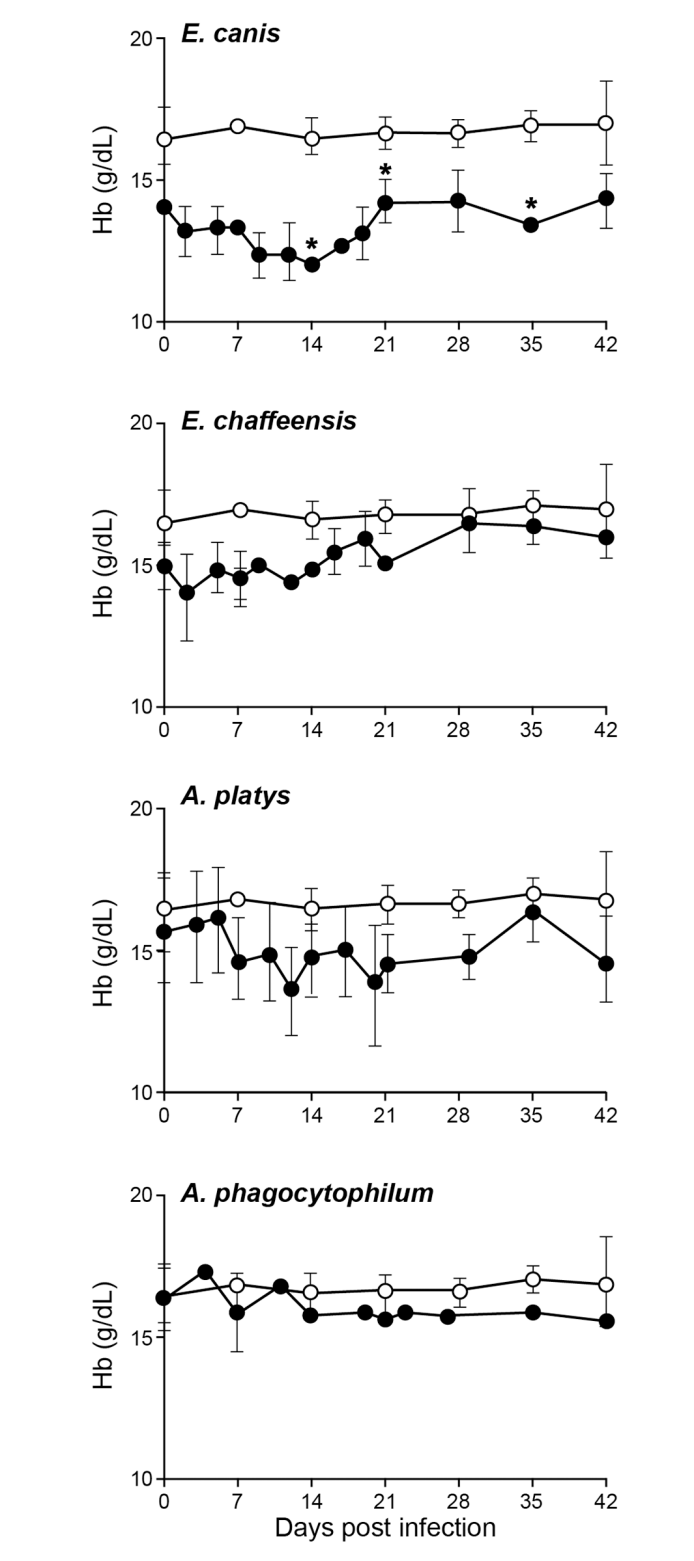
Impact of infections on hemoglobin (Hb) values in dogs. Hb values in uninfected controls are compared to those observed in infected groups. Error bars for this panel represent mean values ±SD for animals within each group. Significant differences (*P* ≤ 0.05) observed in an infected group (closed circle) compared to non-infected control (open circle) values are identified with asterisks.

**Fig 4:**
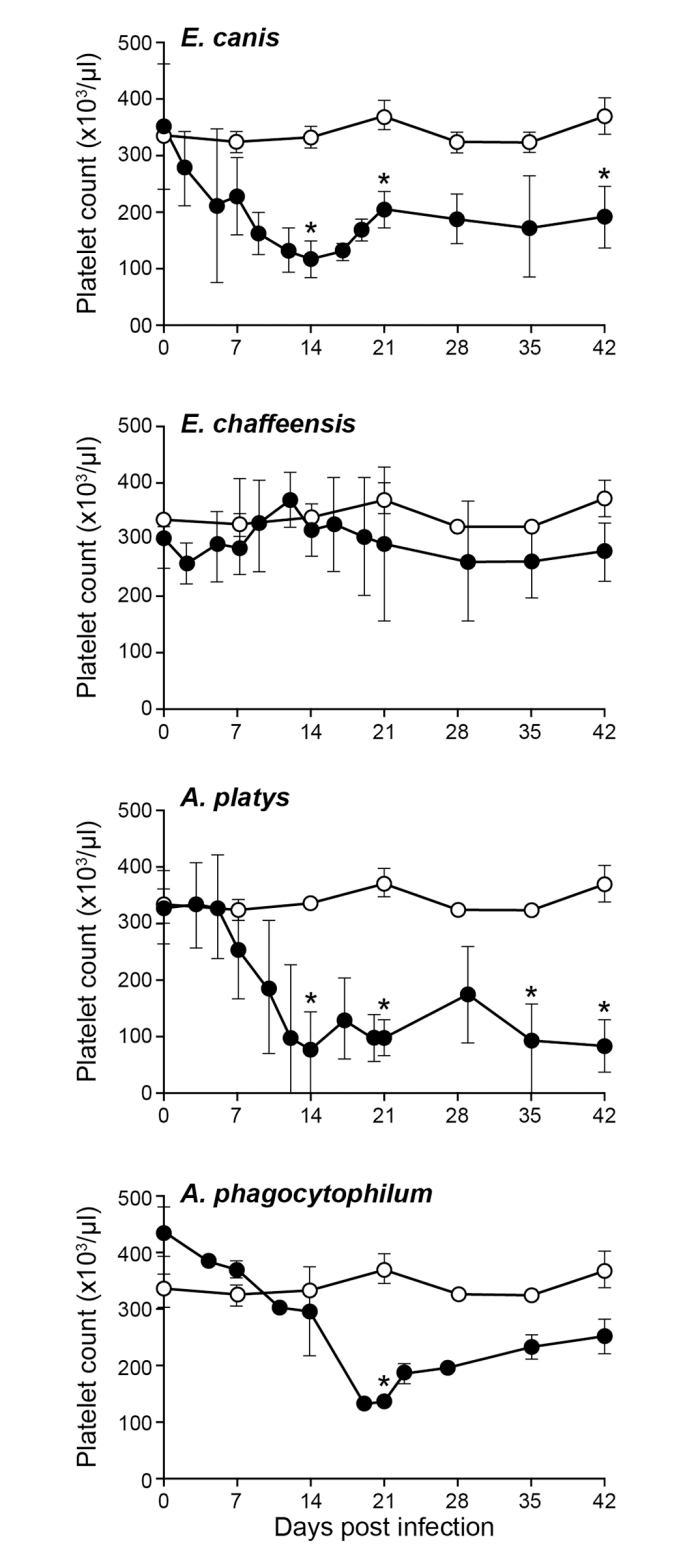
Impact of infections on platelet counts. The platelet counts in dogs infected with *E*. *chaffeensis*, *E*. *canis*, *A*. *platys* and *A*. *phagocytophilum* were compared with the values observed for uninfected controls. The values are shown as mean ± SD per group. Significant differences (*P* ≤ 0.05) observed between controls (open circle) and infected groups (closed circle) are identified with asterisks.

The packed cell volume (PCV), red blood cell counts (RBC), hemoglobin (Hb) and platelet counts were assessed for all four groups (Figs 1–4). The *E*. *canis* infection resulted in the most significant effect on the dogs causing hematological changes. Significant changes in PCV, RBC, Hb and platelet counts were observed on several post infection days compared to the control group; PCV for *E*. *canis* infected dogs was lower on days 14 and 42; RBC counts was lower on days 7, 14 and 35; Hb levels were lower on days 14, 21 and 35 and platelet counts were lower on days 14, 21, and 42. For *A*. *platys*, only platelet counts were significantly different from controls on days 14, 21, 35, and 42. Similarly, for *A*. *phagocytophilum*, only platelet counts differed on day 21. No significant differences were detected between *E*. *chaffeensis* and controls for any hematological parameters, although some declining trends were noted.

A trend in increased total leukocytes counts (TLC) was noted only in *A*. *platys* infected animals (S1 Fig). Increase in neutrophils and monocytes for this group is also observed similarly, as judged from differential leukocyte count analysis (S1 Fig). These increases were not statistically significant from controls as there is a greater variation observed among infected dogs. Interestingly, despite the fact that dogs infected with *E*. *chaffeensis* and *E canis* developed monocytopenia, the TLC remained very similar to the control group throughout the assessment period.

**Fig 5:**
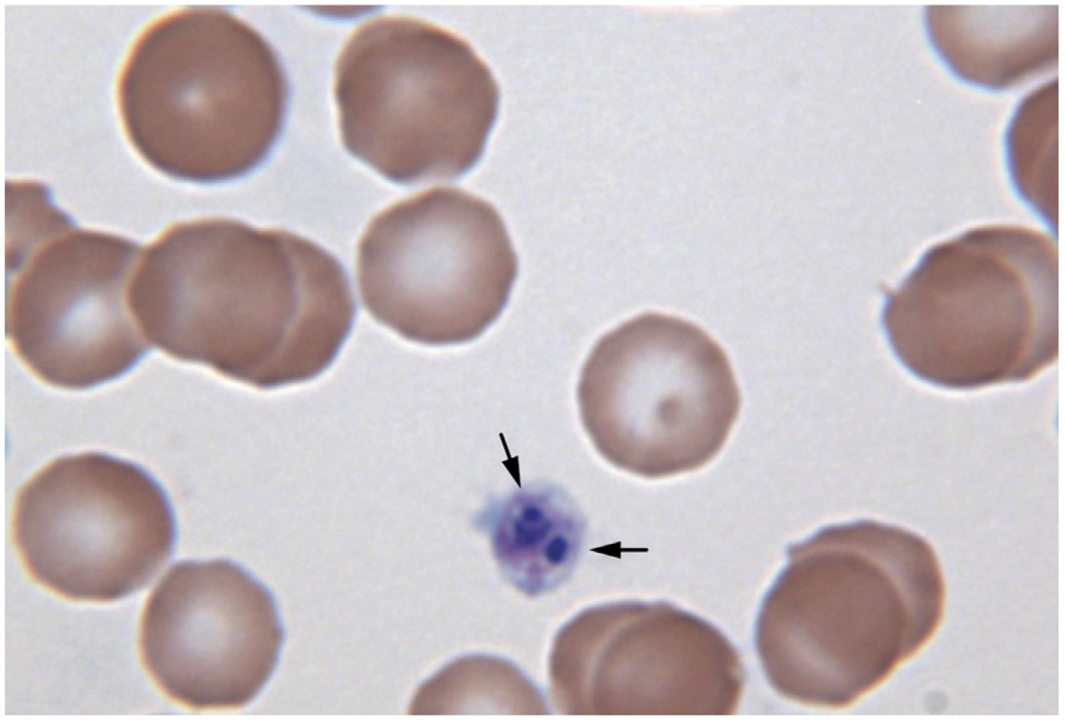
Positives blood smear from an *A*. *platys* infected dog. The inclusions were identified with arrow head as purple stained bodies within the platelet cytoplasm.

Rickettsial morulae were not identified in the blood smears of animals infected with *E*. *canis* or *E*. *chaffeensis*. *A*. *platys* morulae were detected in the blood smears in 3 out of 4 dogs on days 9 to 17 post infection (Fig 5). Blood smear evaluation also revealed the presence of reactive lymphocytes and monocytes in all three infected groups assessed; *E*. *canis*, *E*. *chaffeensis* and *A*. *platys*, but not in the control animals and in the animals prior to infections. Further, blood smear analysis revealed the presence of granular lymphocytes in all days of assessment (days 2 to 19 post infection). The granular lymphocytes in blood was estimated comparing to the TLC. They ranged from 50–600 /μl in *E*. *chaffeensis* infected group. Similarly, *E*. *canis* and *A*. *platys* infected dogs had granular lymphocytes, which ranged from 50–200 granular lymphocytes/μl.

### Pathogen detection by culture and molecular methods

Dog blood samples were monitored once every 3–4 days for the presence of viable organisms by *in vitro* culture analysis for *E*. *canis* and *E*. *chaffeensis* and the presence of all four organisms was also assessed by conventional semi-nested PCR (Table 2). Blood positives for PCR or culture recovery were observed frequently in all four groups of infected dogs for the entire assessment period of 42 days. *E*. *canis* was the most frequently recovered by culture; all four dogs in this group tested positive on every sampling day starting from day 7 (Table 2). The *A*. *platys* infected animal samples were the second most frequently detected PCR positives for the organism in blood (92% of the time) (Table 2). *E*. *chaffeensis* culture positives and *E*. *chaffeensis* and *A*. *phagocytophilum* DNA positives were detected less frequently in blood (75% and 50%, respectively) (Table 2) compared to *E*. *canis* and *A*. *platys*. The presence of genomic DNAs of the pathogens was confirmed in all infected groups at the end point of the study when tissue samples were assessed by PCR (Table 3). DNA of all four pathogens was detected in liver, spleen, cervical lymph node or lung. Spleen or lymph nodes tested PCR positives for all four infected groups and in several animals, whereas liver and lung tested negative the DNA for *A*. *phagocytophilum* and only lung tissues tested negative for *A*. *platys* infected dogs. Also, *E*. *canis* and *E*. *chaffeensis* infected dogs had more PCR positives in tissue samples than *A*. *platys* and *A*. *phagocytophilum* infected groups. All blood samples and all four tissue samples from uninfected control dogs were negative for all four pathogens.

**Table 2:** *Ehrlichia* and *Anaplasma* species infection progression monitored by culture isolation and PCR.

		Infection status by days post infection						
Bacteria	Dog #	0	7	14	21	27	35	42
*E*. *canis*	1	-	+	+	+	+	+	+
	2	-	+	+	+	+	+	+
	3	-	+	+	+	+	+	+
	4	-	+	+	+	+	+	+
*E*. *chafeensis*	1	-	-	+	+	+	-	+
	2	-	+	+	-	+	+	+
	3	-	+	-	+	-	+	+
*A*. *platys*	1	-	+	+	+	+	+	+
	2	-	-	-	+	+	+	+
	3	-	+	+	+	+	+	+
	4	-	+	+	+	+	+	+
*A*. *phagocytophilum*	1	-	-	-	+	-	-	-
	2	-	-	+	+	+	+	+
	3	-	-	+	+	-	-	+
Control	1	-	-	-	-	-	-	-
	2	-	-	-	-	-	-	-

The signs − and + refer to samples tested negative or positive by culture recovery and/or by molecular method(s).

**Table 3:** Infection status of tissue samples in dogs infected with *E*. *canis*, *E*. *chaffeensis*, *A*. *phagocytophilum* and *A*. *platys*.

	Infection status by group and animal number															
	*E canis*				*E*. *chaffeensis*			*A*. *platys*				*A*. *phagocytophilum*			Control	
Tissues	1	2	3	4	1	2	3	1	2	3	4	1	2	3	1	2
Spleen	+	+	+	+	-	+	+	+	+	+	-	+	-	-	-	-
Lymph node	+	+	+	+	+	+	+	-	-	-	+	+	-	-	-	-
Liver	+	-	-	+	+	+	-	-	-	+	-	-	-	-	-	-
Lung	-	-	+	+	-	-	+	-	-	-	-	-	-	-	-	-

The − and + signs refer to samples tested negative or positive by nested PCR, respectively.

### IgG response in infected dogs

**Fig 6:**
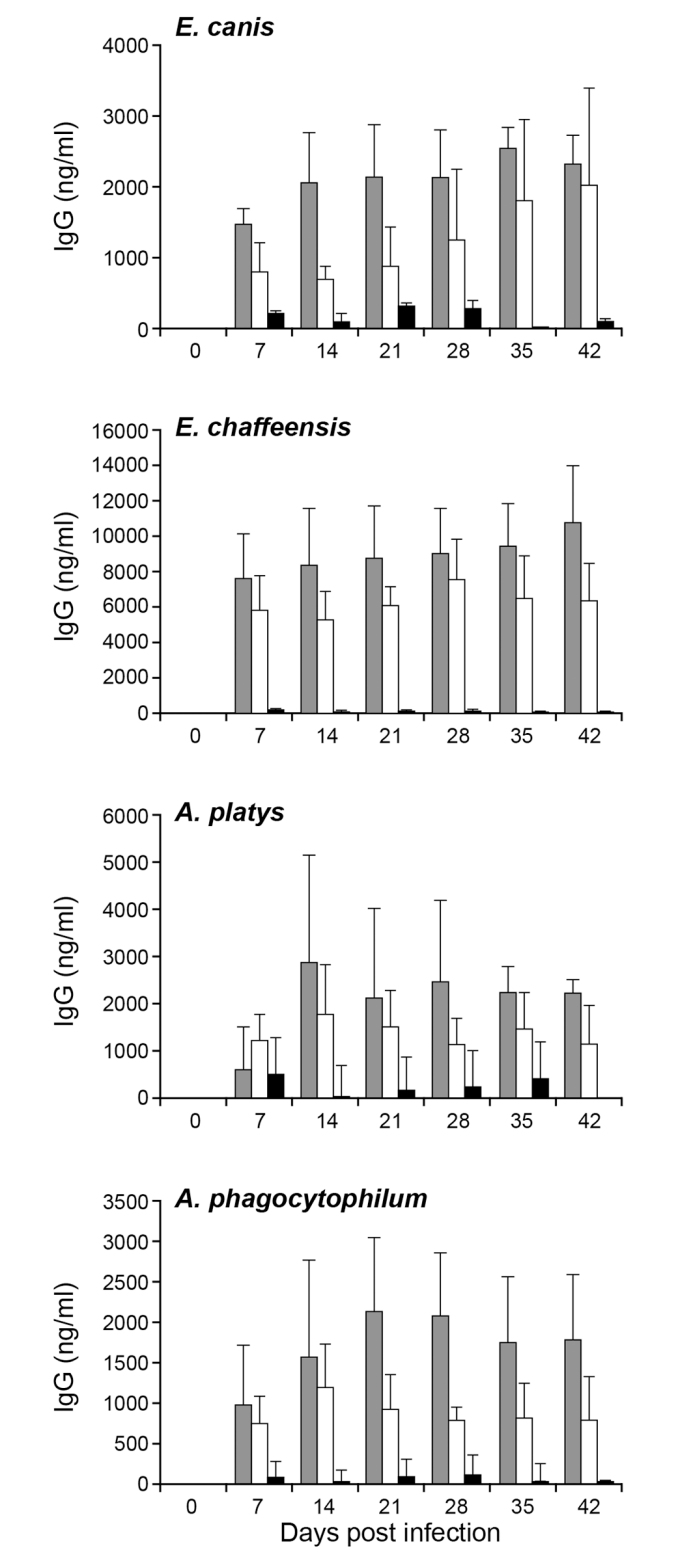
Total IgG and IgG subclass response in dogs following the pathogens’ infections. Plasma samples were assessed to measure the concentrations of total IgG and IgG subclasses, IgG1 and IgG2 respectively. The quantity of each IgG was determined by comparing the absorbance observed for known quantities of positive controls. The values were presented as bars representing mean values ± SD. (Total IgG, gray bars; IgG1 black bars; IgG2 open bars).

Total IgG and IgG subclasses (IgG1 and 2) were evaluated by quantitative ELISA. All infection groups developed pathogen specific IgG responses with predominant expression of IgG2 for all post infection days evaluated (Fig 6). Mean quantity of IgG2 in each group was significantly higher than IgG1 for every sample assessed (P<0.001). *E*. *chaffeensis* infection in dogs induced the highest antibody production, while the remaining three pathogen infections resulted in similar amounts of antibody production per ml of blood.

### Necropsy, Histopathology and tissue sample analysis

**Fig 7:**
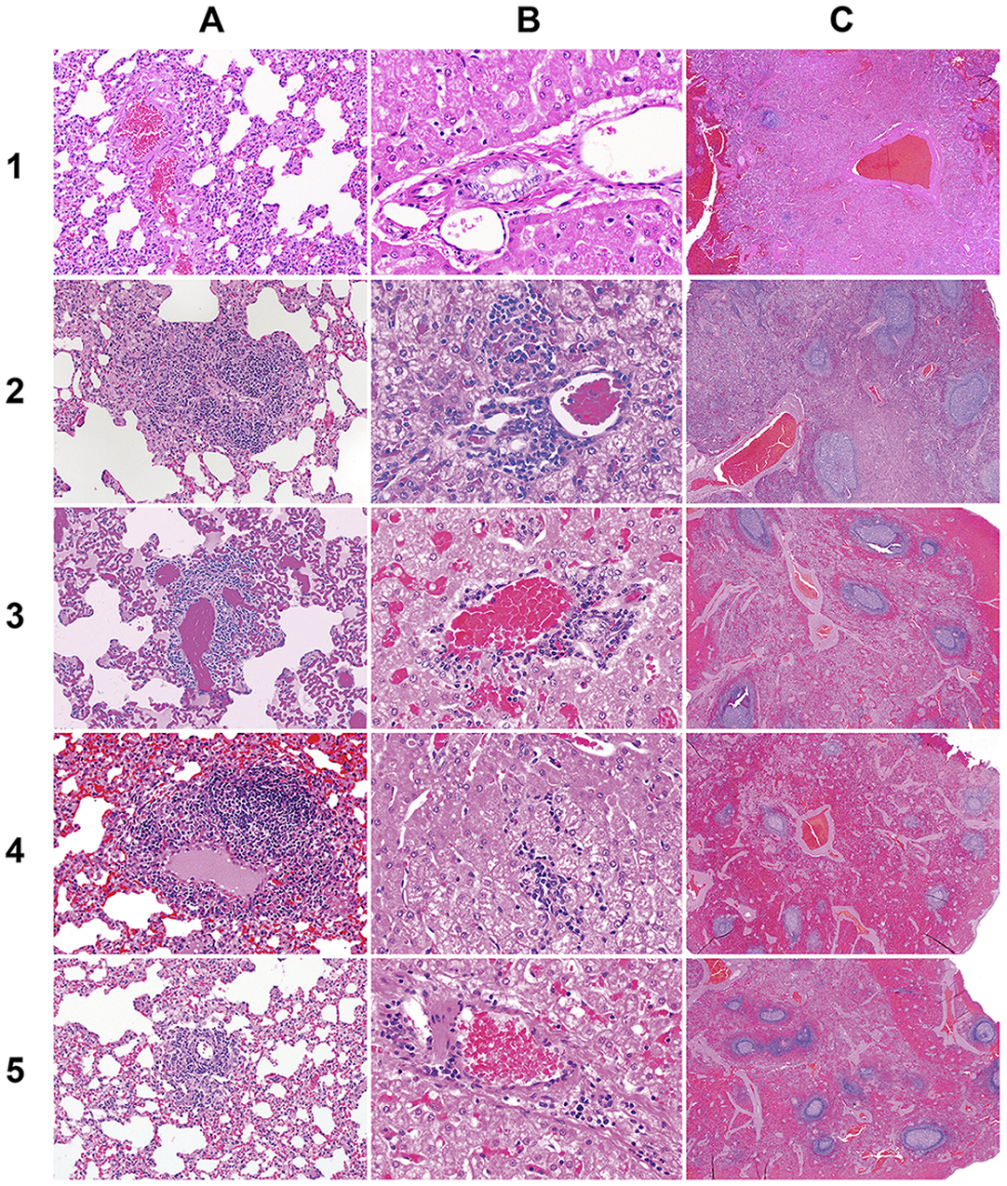
Histopathological changes in dogs resulting from infections. Rows 1–5 represent tissue samples collected from controls, *E*. *canis*, *E*. *chaffeensis*, *A*. *platys* and *A*. *phagocytophilum* infected dogs, respectively. A) Lung tissues showing perivascular infiltration of macrophages and lymphocytes with variation in the degree of inflammation among infected groups compared to controls. B) Liver tissues having periportal infiltrates of lymphocytes and macrophages with variation in the degree of inflammation observed among infected groups compared to controls. C) Spleen tissues showing periarteriolar lymphoid sheet (white pulp) hyperplasia with variation in the size of the white pulp in infected groups compared to controls. One section each of randomly selected samples with magnifications of 200x for lung, 400x of liver and 20x of spleen were presented.

**Fig 8:**
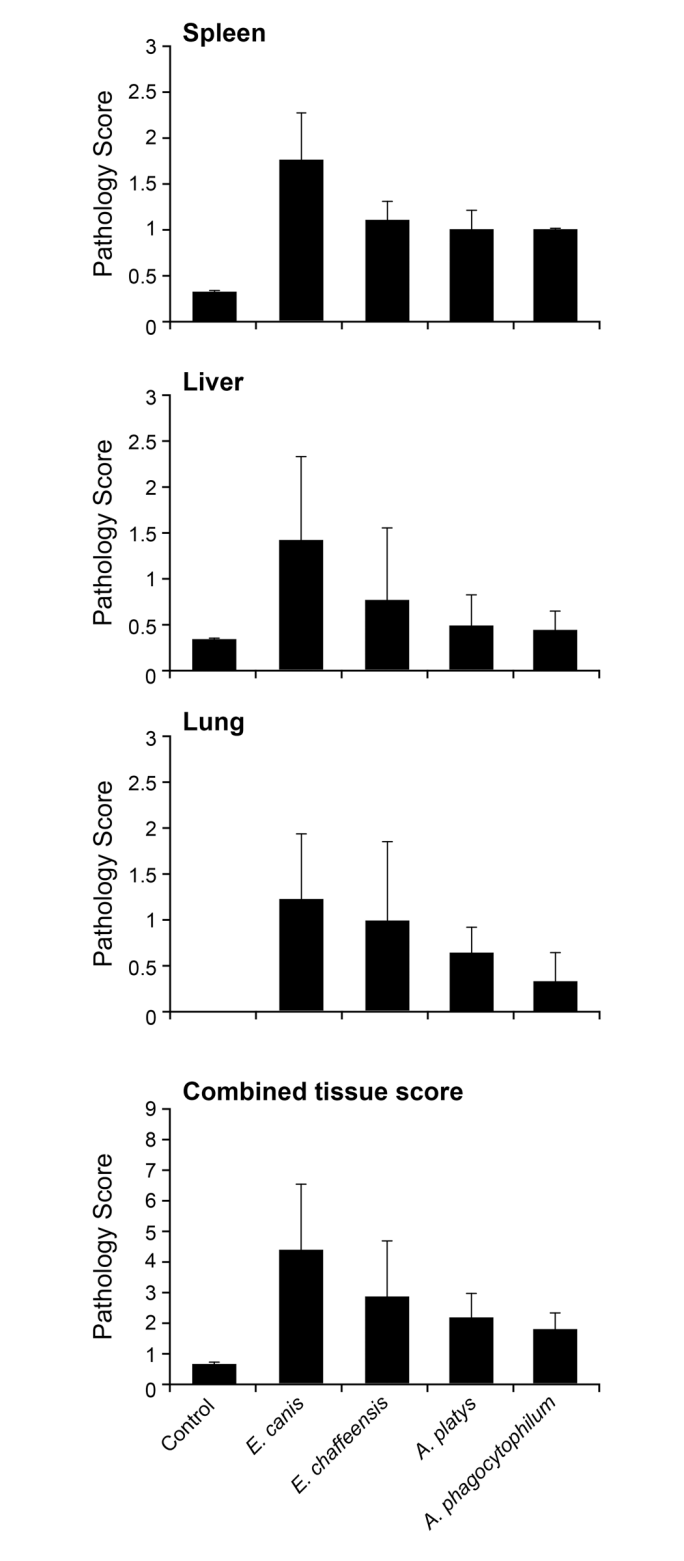
Histopathological grading scores of lung, liver,spleen tissues and combined average values. Histopathological observations of tissue samples from each dog with assigned numerical scores were presented for spleen, liver, lung and for the combined average values for all three tissues. Bar represent mean scores ± SD within each group.

With the exception of splenomegaly observed in one dog infected with *E*. *canis*, no other gross lesions were noticed in any of the infected animals. Histopathological analysis of lung, liver and spleen revealed pathological changes in all four pathogen infected groups, while the tissue samples from control animals had no microscopic lesions (Figs 7 and 8). Lung tissues of the *E*. *canis* infected dogs had scattered to poorly delineated or discrete microgranulomas. Similar observations were noticed in dogs infected with *A*. *platys* (Fig 7A). The inflammation was characterized by moderate to large number of perivascular infiltrates of macrophages and lymphocytes in lungs of the *E*. *canis* infected dogs, while the *E*. *chaffeensis* and *A*. *platys* infected dogs had small to moderate numbers of macrophages and lymphocytes. *A*. *phagocytophilum* infected group revealed only mild inflammatory changes in lungs. In the *E*. *canis* infected group, liver lesions revealed moderate periportal infiltrates of lymphocytes and macrophages with occasional sinusoidal microgranulomas. One animal in the *E*. *chaffeensis* group exhibited mild periportal and sinusoidal microgranulomas. Mild periportal inflammatory lesions were observed in the liver sections of *A*. *phagocytophilum* and *A*. *platys* infected animals (Fig 7B). Mild to moderate lymphoid hyperplasia was evident in spleens of *E*. *canis*, *E*. *chaffeensis* and *A*. *platys* infected animals, whereas only mild lymphoid hyperplasia was observed in *A*. *phagocytophilum* infected animals (Fig 7C). Pathological grading revealed minor variations in animals within each infected group; pathological changes were the highest for the *E*. *canis* infected group, followed by *E*, *chaffeensis*, *A*. *platys* and *A*. *phagocytophilum*, respectively (Fig 8).

## Discussion

The primary goal of the current study was to compare infection progression in dogs with the commonly reported tick-borne pathogens of the family *Anaplasmataceae*; namely *E*. *canis*, *E*. *chaffeensis*, *A*. *platys* and *A*. *phagocytophilum*. The inocula used for infecting dogs were derived from *in vitro* cultures for *E*. *chaffeensis*, *E*. *canis* and *A*. *phagocytophilum* or from infected dog blood (*A*. *platys*) and infections were carried out as IV inoculations. While it is ideal to compare infection progressions via tick transmission, it is difficult to accomplish this task due to technical limitations. The IV method is a viable option, as most experimental infections are carried by this method, thus allowing comparisons with prior published research. During feeding, ticks deposit pathogens into a feeding lesion created by the mechanical action of the chelicerae and the hypostome, and by the activity of their highly complex saliva that is composed of potent anti-inflammatory, immune-modulatory and anti-hemostatic factors. These create an environment that protects the pathogen from host defenses [25,26]. By contrast, intradermal injection places pathogens into an immune-competent site, which greatly diminishes their ability to infect the host and limits dissemination to target organs [27]. Because tick-transmission models are not available for *A*. *platys*, we chose to use IV inoculation for all pathogens for consistency. In support of this approach, we recently described *E*. *chaffeensis* infections in deer by two different modes of infection (IV and tick transmission). We also assessed the infections in deer and dogs with IV inoculum prepared from the organisms cultured in tick cells or in macrophage cultures [28]. Independent of the methods of inoculation or the source of the organisms, both deer and dogs developed similar persistent infections, although there were some differences noted in the IgG antibody responses. Independent of variations in the sources of inocula, results of the current study reveal that all four pathogens cause persistent infection in dogs with minor variable hematological and pathological outcomes.

Infection with *E*. *canis* resulted in more pronounced hematological changes, such as reduced PCV and anemia. The anemia observed in the current study is similar to prior documentation for *E*. *canis* infections occurring naturally or experimentally [13,29]. These hematological abnormalities, however, cannot serve as indicators for *E*. *canis* infection, as *A*. *platy*s infection also caused a similar drop in PCV. Dogs infected with the platelet-trophic *A*. *platys* manifested severe thrombocytopenia starting from day 10 after infection. This abnormal platelet value continued throughout the 42 days of assessment. The drop in platelet count is similar to previous reports [4,29,30]. However, the cyclical thrombocytopenia previously reported with *A*. *platys* infection was not observed in the present study. Interestingly, persistent thrombocytopenia was also observed with *E*. *canis* and *A*. *phagocytophilum* infections even though cell tropisms for these pathogens are monocytes and granulocytes, respectively. A trend towards depressed platelet counts was also noted for *E*. *chaffeensis* infection in the current study, which is similar to prior data [15]. Thrombocytopenia resulting from *A*. *platys* infection is considered to be a result of infected platelets, whereas *E*. *canis* infection is thought to depress thrombocytes by producing anti platelet antibodies [31–33]. Thrombocytopenia with *A*. *phagocytophilum* infection has likewise been reported earlier [34]. Importantly, the major hematological change observed in dogs infected with all four pathogens was thrombocytopenia and this abnormality did not serve as a guide to distinguish the four tick-borne infections.

Fever is one of the most common clinical signs associated with ehrlichiosis and anaplasmosis. Fever was observed more frequently in dogs infected with both the *Ehrlichia* pathogens and was less common for *Anaplasma* species infections. Only one dog infected with *A*. *platys* developed fever, while none from the *A*. *phagocytophilum* infected group were pyrexic. Although pyrexia has been reported in dogs naturally infected with *A*. *phagocytophilum* [34], strain-specific variations in clinical signs in *A*. *phagocytophilum*-infected dogs are known [16]. The lack of pyrexia for *A*. *phagocytophilum* infected dogs in the present study may represent a strain-specific occurrence. It is also possible that this group of dogs too had transient fever, which we may have missed detecting it due to our sampling frequency of once every 3 days.

As noted by others [35], we were unable to detect inclusions in *E*. *canis* and *E*. *chaffeensis* in canine blood smears despite the persistence of all four pathogens as evidenced by molecular detection methods on different days post infection and/or by culture recovery methods. The only smear positives detected were in dogs infected with *A*. *platys*. Blood smear analysis is the most insensitive method for detecting these pathogens, as reported earlier for *E*. *canis* [36]. Granular lymphocytosis up to 50% is previously documented in dogs with chronic ehrlichiosis [37,38]. In the current study, we also detected the presence of granular lymphocytes in infected dogs for the first 19 days of assessment following infection, which ranged from 50–600 granular lymphocytes/μl (about 4–16% of the total lymphocytes). This may be due to the difference in acute and chronic nature of infection. Prolonged persistence appears to be a common feature for *Ehrlichia* and *Anaplasma* species infections [14–16,30,39,40]. Persistent infections documented in our study are clear evidence that that dogs fail to completely clear the systemic infections with the pathogens. Persistent infection is detrimental to the host, while offering a distinct advantage for the pathogens to continue their life cycle successfully by maximizing the opportunities for reacquisition by ticks.

Pathogen specific total IgG responses were detected in all dogs infected with the four different pathogens starting from day 7, and the antibody was predominantly the Th1 type IgG subclass antibody, the IgG2. The Th1 type IgG response observed here against all four pathogens in dogs is similar to prior observations in mouse infection studies with *E*. *chaffeensis* [24,41]. The current study suggests that despite the high antibody response, it is not sufficient to clear the pathogens from circulation. The data we reported here in the canine host is similar to the prior evidence documented in the murine host model for *E*. *chaffeensis* [42,43].

The major histopathologic change observed in all four groups of infected dogs was the infiltration of perivascular mononuclear cell infiltration into lung, liver and spleen. The degree of infiltration and inflammation varied for each pathogen and the most severe lesions were observed with *Ehrlichia* species compared to *Anaplasma* species. Consistent with these observations, a severe pathology with *E*. *canis* infection in a dog is reported earlier [29,44]. Likewise, HME patients develop lesions, such as multifocal necrosis in liver and lymphoid tissues, interstitial pneumonia, and perivascular lymphohistocytic infiltration [45–47]. Pathologic changes in HGA, caused by *A*. *phagocytophilum* infections, include perivascular lymphohistiocytic inflammatory infiltrates in multiple organs and hyperplasia of spleen and lymph nodes. Such pathologic changes were not observed in dogs in the current study, possibly due to host-specific differences or pathogen strain-specific variations.

## Conclusions

In summary, this is the first study to compare clinical, hematological, and pathological changes in dogs resulting from infections with four closely related tick-borne rickettsial pathogens. The study demonstrated that despite minor pathogen-specific variations noted, the infections are difficult to be distinguished from clinical or hematological observations. Infections are also difficult to be monitored by blood smear or antibody analysis, due to low sensitivity and extensive serological cross-reactions [36,48–51], as whole cell antigen-based antibody tests cannot identify the causative agents. Infection persistence with all four pathogens, observed in this study, is an added complication for these tick-borne pathogens.

## Supporting Information

S1 Fig Impact of *A*. *platys* infection on leukocyte counts. TLC, neutrophils, lymphocytes and monocytes in dogs infected with *A*. *platys* were compared with the values observed for uninfected controls. The values are shown as mean ± SD per group.
(TIFF)
