# Supplementary figures and images for: Comparative Experimental Infection Study in Dogs with Ehrlichia canis, E. chaffeensis, Anaplasma platys and A. phagocytophilum
Source: PLoS One. 2016 Feb 3;11(2):e0148239. doi: 10.1371/journal.pone.0148239 (PMC4739612; doi:10.1371/journal.pone.0148239)

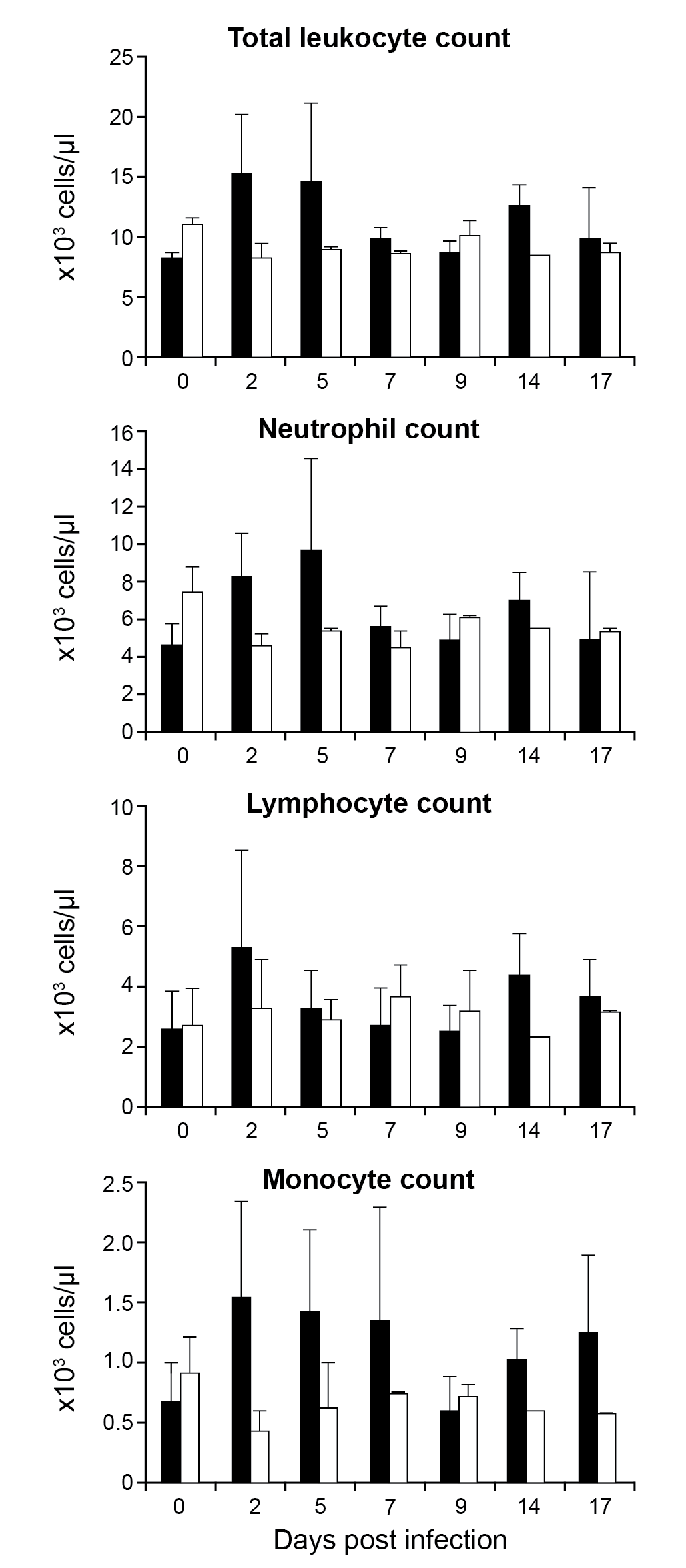

Supplement: S1 Fig — TLC, neutrophils, lymphocytes and monocytes in dogs infected with A. platys were compared with the values observed for uninfected controls. The values are shown as mean ± SD per group. (TIFF) [file pone.0148239.s001.tiff]
